# Contributions of amino acid, acylcarnitine and sphingolipid profiles to type 2 diabetes risk among South-Asian Surinamese and Dutch adults

**DOI:** 10.1136/bmjdrc-2019-001003

**Published:** 2020-05-05

**Authors:** Mirthe Muilwijk, Susan M I Goorden, Carlos Celis-Morales, Michel H Hof, Karen Ghauharali-van der Vlugt, Femke S Beers-Stet, Jason M R Gill, Frédéric M Vaz, Irene G M van Valkengoed

**Affiliations:** 1 Department of Public Health, Amsterdam Public Health Research Institute, Amsterdam UMC, University of Amsterdam, Amsterdam, The Netherlands; 2 Laboratory Genetic Metabolic Diseases, Amsterdam UMC, University of Amsterdam, Amsterdam, The Netherlands; 3 BHF Glasgow Cardiovascular Research Centre, Institute of Cardiovascular and Medical Sciences, University of Glasgow, Glasgow, UK; 4 Department of Clinical Epidemiology, Biostatistics and Bioinformatics, Amsterdam UMC, University of Amsterdam, Amsterdam, The Netherlands

**Keywords:** metabolism, epidemiology, ethnic differences, type 2 diabetes

## Abstract

**Introduction:**

People of South Asian origin are at high risk of type 2 diabetes (T2D), but the underpinning mechanisms are not fully understood. We determined ethnic differences in acylcarnitine, amino acid and sphingolipid concentrations and determined the associations with T2D.

**Research design and methods:**

Associations between these metabolites and incident T2D among Dutch and South-Asian Surinamese were determined in participants from the Healthy Life in an Urban Setting (HELIUS) study (Amsterdam, the Netherlands) using Prentice-weighted Cox regression. The HELIUS study includes 95 incident T2D cases and a representative subcohort of 700 people from a cohort of 5977 participants with a mean follow-up of 4 years.

**Results:**

Concentrations of acylcarnitines were comparable between both ethnic groups. Amino acid and lactosylceramide concentrations were higher among South-Asian Surinamese than Dutch (eg, isoleucine 65.7 (SD 16.3) vs 60.7 (SD 15.6) µmol/L). Ceramide concentrations were lower among South-Asian Surinamese than Dutch (eg, Cer d18:1 8.48 (SD 2.04) vs 9.08 (SD 2.29) µmol/L). Metabolic dysregulation preceded T2D without evidence for a multiplicative interaction by ethnicity. Most amino acids and (dihydro)ceramides were associated with increased risk (eg, Cer d18:1 HR 2.38, 95% CI 1.81 to 3.12) while acylcarnitines, glycine, glutamine and lactosylceramides were associated with decreased risk for T2D (eg, LacCer d18:2 HR 0.56, 95% CI 0.42 to 0.77).

**Conclusions:**

Overall, these data suggest that the disturbances underlying amino acid and sphingolipid metabolism may be predictive of T2D risk in populations of both South Asian and European background. These observations may be used as starting point to unravel the underlying metabolic disturbances.

**Significance of this study**

**What is already known about this subject?**

- People of South Asian descent are at a twofold to fourfold increased risk of type 2 diabetes (T2D) compared with people of European descent.

**What are the new findings?**

- Concentrations of metabolites associated with T2D differ between South Asians and Europeans.
- In both Dutch and South-Asian Surinamese, higher plasma concentrations of certain acylcarnitines, glycine, glutamine and lactosylceramides decreased T2D risk, while higher plasma concentrations of (dihydro)ceramides and most amino acids increased T2D risk.
- The ethnic differences in amino acid and lactosylceramide concentrations could potentially contribute to the higher T2D risk among South-Asian Surinamese compared with Dutch; however, ceramide profiles are more beneficial among South-Asian Surinamese.

**How might these results change the focus of research or clinical practice?**

- The results may be used as starting point to unravel ethnic differences in T2D risk by identifying underlying metabolic disturbances.
- Plasma amino acid and sphingolipid concentrations may be used as markers to identify people at risk for T2D.

## Introduction

More than 425 million people are affected by type 2 diabetes (T2D) worldwide, and this number is expected to grow to 629 million by 2045.^1^ The incidence of T2D is two to four times higher among South Asian compared with people of European descent.^2 3^ The etiological mechanisms behind the ethnic differences in T2D risk are not fully understood. One potential contributor to these differences is lipid overload, which can lead to increased incomplete fatty acid oxidation, mitochondrial stress,^4 5^ and also to ectopic fat accumulation causing an increased use of non-oxidative pathways.^6^ These processes can be assessed by measuring circulating concentrations of branched-chain amino acids (BCAA) and acylcarnitines, which both serve as markers of mitochondrial stress,^4 5^ and circulating ceramides which may signify the use of non-oxidative pathways.^6^ Evidence exists that some sphingolipids, acylcarnitines, and BCAA and aromatic amino acids are associated with T2D.^7–9^ However, for other amino acids and many types of sphingolipids, there is very limited evidence of an association with T2D. Furthermore, it is unclear whether ethnic differences in these metabolite concentrations potentially contribute to the higher risk of T2D in South Asian compared with European populations. Therefore, it is necessary to determine the metabolite concentrations and identify the associations with T2D.

There is some limited evidence that both plasma concentrations of amino acids and acylcarnitines affect the risk of T2D,^5 10 11^ and that the strengths of these associations differ between ethnic groups.^5 12^ To add to the limited evidence in base, we therefore aimed to investigate the potential contribution of amino acids, acylcarnitines and sphingolipids to the high risk for T2D observed among South Asians. To do this, we used data from the Healthy Life in an Urban Setting (HELIUS; Amsterdam, the Netherlands). The results of our study will contribute to insights in mechanisms that are potentially important in the pathophysiology of T2D in diverse populations. Furthermore, it may add to efforts to identify persons who are at increased risk to develop T2D in these groups.

This study had three specific objectives. First, to quantify baseline acylcarnitine, amino acid and sphingolipid plasma concentrations among Dutch and South-Asian Surinamese; second, to quantify the association between metabolites (acylcarnitines, amino acids and sphingolipids) and incident T2D; and third, to determine whether the strengths of associations are similar in Dutch and South-Asian Surinamese. Previous work has shown that, although absolute risks between people living in various countries may differ, relative differences in cardiovascular disease risk factors between ethnic groups are similar to other European countries, suggesting that our results are generalizable to other European countries.^13^

## Research design and methods

### Population

We used baseline data from the HELIUS study, which were collected between 2011 and 2015. HELIUS is a multiethnic cohort study among six ethnic groups living in Amsterdam. A detailed description of the design is available elsewhere.^14 15^ In brief, we randomly sampled participants from the municipal register, stratified by ethnicity. Questionnaires, physical examinations, and biological samples were obtained.^15^

**Figure 1:**
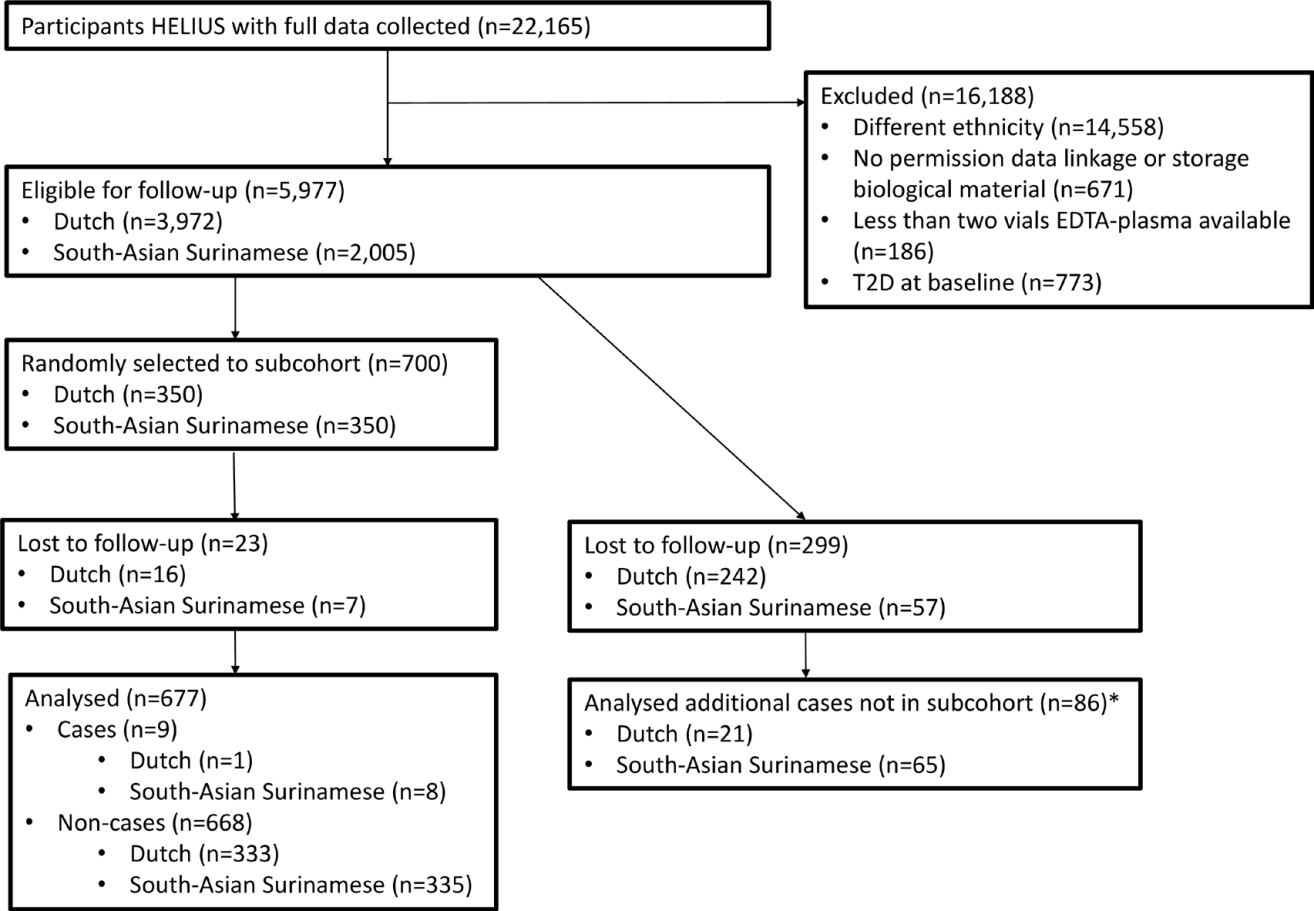
Consolidated Standards of Reporting Trials (CONSORT) diagram of the inclusion of participants. A random sample of 350 participants per ethnic group was taken from the eligible population and included as the subcohort for whom metabolites were measured. In addition, metabolites were measured for all incident T2D cases. *A total of 95 cases developed T2D of which nine were in the subcohort. HELIUS, Healthy Life in an Urban Setting; T2D, type 2 diabetes.

A case–cohort design was used to increase efficiency.^16^ Use of a case–cohort design can provide unbiased estimates, without losing much precision compared with analyzing the full cohort.^16^ For this study, a random sample of 350 participants of Dutch and 350 participants of South-Asian Surinamese ethnicity was selected as the subcohort (control group). We focused on South-Asian Surinamese because we aimed to unravel the potential underlying causes for the high risk of T2D of South Asian origin populations compared with European origin populations. The Dutch participants were included as reference populations, since this ethnic group is the host population and at particular low risk for T2D.^2 3^ Acylcarnitines, amino acid and sphingolipid concentrations were determined for this subcohort, and for all participants who developed T2D (cases; figure 1).

### Measurements

Incident T2D cases were identified through record linkage with two healthcare registrations (online supplementary material S1). We first linked HELIUS data by Citizen Service number to the Achmea Health Database with registrations from 1 January 2010 until 30 April 2016. Second, HELIUS data were linked to Vektis with probabilistic linkage based on data of birth, sex and postal code, with registrations from 1 January 2011 until 31 December 2017. We defined incident T2D as a registration of one of the considered codes in either one of the databases, and not having T2D at baseline based on self-report, medication use, glucose or HbA1c levels. Follow-up duration was determined from inclusion date within HELIUS until the moment of data linkage, or the year that a participant developed T2D.

10.1136/bmjdrc-2019-001003.supp1 Supplementary data

Ethnicity was defined by the individual’s country of birth combined with the parental countries of birth. Dutch ethnicity was assigned to participants born in the Netherlands, with both parents born in the Netherlands. South-Asian Surinamese ethnicity was assigned to participants born in Suriname with at least one parent born in Suriname (first generation) or born in the Netherlands with both parents born in Suriname (second generation) combined with self-reported South Asian ethnic origin.^15^

Information on pack-years of smoking and physical activity was determined from the questionnaire. The number of pack-years was calculated by multiplying the number of packs (containing 20 cigarettes) smoked a day by the number of years. Smoking cigars and pipe tobacco was also included by calculating the equivalent rates of tobacco. The physical activity score was derived by the Short Questionnaire to Assess Health (SQUASH) enhancing physical activity, which includes questions on activities at work and school, leisure time, household activities, commuting activities and other daily activities and the intensity at which the activity was executed.^17^ Results of the SQUASH enhancing physical activity were converted to minutes per week and multiplied by the metabolic equivalent (MET) intensity score. Body mass index (BMI) was determined by dividing measured body weight (kg) by height squared (m^2^). Weight and height were measured in barefoot subjects wearing light clothes only. Waist circumference was measured using a tape measure at the level mid-way between the lowest rib margin and the iliac crest. All anthropometric measures were taken in duplicate and the mean was used in the analyses. If the discrepancy between the duplicate measures differed more than 0.5 cm for height, more than 0.5 kg for weight or more than 1 cm for waist circumference, a third measurement was taken. The two measures which were most similar were used to calculate the mean.

### Laboratory methods

Blood was collected after a fasting period of at least 10 hours. Acylcarnitines and amino acids were determined in plasma by tandem-mass spectrometry as described previously.^18 19^ (Glyco)sphingolipids were determined using the following chemicals and reagents: sphingosine (d18:1), sphinganine (d18:0), 1-deoxysphinganine (m18:0), glucosylsphingosine (d18:1), lactosylsphingosine (d18:1), and d5-glucosylsphingosine (d18:1) were from Avanti Polar Lipids and all organic solvents were from Biosolve (liquid chromatography-tandem mass spectrometry (LC-MS/MS) quality). Formic acid, butanol, and hydrochloric acid were obtained from Merck and globotriaosylsphingosine (d18:1), sodium hydroxide, and ammonium formate were from Sigma. Samples were prepared for analysis by extraction of (glyco)sphingolipids from plasma with chloroform and methanol. In short, 50 µL plasma was pipetted in a 2 mL tube and 400 µL methanol and 200 µL chloroform were added. Extract was transferred to a 2 mL tube and separation of phases was induced by addition of 310 µL water. The lower phase was transferred to a Pyrex tube and the upper phase was washed with chloroform. Next, combined lower phases, containing (glyco)sphingolipids, were dried under a stream of nitrogen and the residue was dissolved in 500 µL 0.1 M sodium hydroxide in methanol. Samples were subjected to microwave-assisted deacylation and neutralized by addition of 1 M hydrochloric acid in methanol. Internal standard (d5-glucosylsphingosine (d18:1)) was added to each sample followed by evaporation of methanol (N_2_, 40°C). Thereafter, samples were subjected to extraction with butanol:water (1:1, v/v). After drying the butanol phase, samples were dissolved in 120 µL methanol and analyzed by LC-MS/MS. For that lyso(glyco)sphingolipids were separated by reversed-phase ultraperformance liquid chromatography (UPLC) using an Acquity I-Class UPLC with ethylene bridged hybrid C18 column, 2.1×50 mm with 1.7 µm particle size (Waters) and detected by electron spray ionization in positive mode and MS/MS instrument (Xevo TQ MS, Waters) in multiple reaction monitoring mode (online supplementary material S2).^20^ Levels of (glyco)sphingolipids were calculated using calibration lines within the appropriate concentration range, according to the internal standard ratio method.

### Statistical analyses

All analyses were conducted in RStudio V.0.99.903.^21^ Metabolites with more than 5% of the data below the detection limit were excluded from further analyses as imputation may lead to inaccuracies.^22^ For other metabolites with measurements below the detection limit, concentrations of half the detection limit were imputed. Finally, outliers for glycine (n=1), serine (n=1) and asparagine (n=1) were set to the median concentration.

We first examined baseline characteristics and quantified metabolite concentrations in the subcohort, as the subcohort represented the entire population. We calculated means and SD for continuous normally distributed variables, medians and IQRs for continuous non-normally distributed variables and numbers of observations and percentages for categorical variables.

Second, we inspected if it was realistic to assume that the metabolites were normally distributed by plotting histograms and checking skewness and kurtosis. Since some metabolites followed a highly skewed distribution metabolites were log10 transformed and z-score standardized before further analysis; after transformation, all metabolites followed a normal distribution. Second, we used a summary score of the metabolites conducting a principal component analysis (PCA). We used the subcohort to construct the PCA and determined individual scores of cases with these factor loadings. We quantified the association between metabolites and incident T2D by weighted Cox proportional hazards regression models using the Prentice method to account for the case–cohort design. HRs for the association between metabolites and incident T2D were reported. Furthermore, to determine whether the strengths of associations are similar in Dutch and South-Asian Surinamese we checked the interaction between metabolites and ethnicity with incident T2D as the outcome. The interaction between sex and metabolites was checked as well, but since none was observed we did not stratify our analyses by sex.

The models were adjusted for the confounders sex, age, smoking, physical activity, BMI and waist circumference, and where relevant ethnicity. This was in accordance with our conceptual model, published at dagitty.net/mrZnVAV. As a sensitivity analysis we additionally adjusted for socioeconomic status.

P values <0.05 were regarded as statistically significant. To avoid type 1 errors we characterized analyses with the principal components (PCs) as the main outcomes, and considered individual metabolites as secondary outcomes. Glutamate and glutamine, and asparagine and aspartate can convert into each other in the samples. We therefore also examined these metabolites combined. We adjusted analyses with individual metabolites for multiple testing by Holm adjustment.

## Results

### Baseline characteristics and metabolite concentrations

The mean age in the subcohort was 45.0 (SD 13.4) years old, with Dutch participants somewhat older than the participants of South-Asian Surinamese origin (figure 1 and table 1). The random subcohort consisted of more women than men (53.3%), especially among South-Asian Surinamese (56.0%). Median pack-years of smoking was 0.1 (IQR 0.0; 6.5), mainly due to very low prevalence of smoking among South-Asian Surinamese participants. Mean physical activity level was 2781 (SD 1578) MET/week, with the Dutch being somewhat more active than South-Asian Surinamese. Mean BMI of the subcohort was 25.1 (SD 4.1) kg/m^2^, and mean waist circumference was 89.4 (SD 12.3) cm. These measures of adiposity were somewhat higher among South-Asian Surinamese than among the Dutch. Most participants (44.6%) were highly educated, while 7.4% of the participants had never been to school or only had elementary schooling. The Dutch were more highly educated than South-Asian Surinamese.

**Table 1:** Baseline characteristics in the subcohort* representative of the entire study population

	Total subcohort (n=677)	Dutch subcohort (n=334)	SA subcohort (n=343)
Mean age (years)	45.0 (13.4)	46.0 (13.8)	44.0 (12.9)
% male (n)	46.7 (316)	49.4 (165)	44.0 (151)
Median pack-years of smoking	0.1 (0.0; 6.5)	1.2 (0.0; 10.0)	0.0 (0.0; 2.8)
Mean physical activity (MET/week)	2781 (1578)	2812 (1312)	2751 (1802)
Mean BMI (kg/m^2^)	25.1 (4.1)	24.4 (3.8)	25.7 (4.4)
Mean waist circumference (cm)	89.4 (12.3)	88.9 (12.2)	90.0 (12.3)
Socioeconomic status (%)			
Never been to school/elementary schooling only	7.4 (50)	4.2 (14)	10.5 (36)
Lower vocational schooling or lower secondary schooling	21.8 (148)	10.8 (36)	32.7 (112)
Intermediate vocational schooling or intermediate/higher secondary schooling	26.1 (177)	21.9 (73)	30.3 (104)
Higher vocational schooling or university	44.6 (302)	63.2 (211)	26.5 (91)

*Baseline characteristics of the subcohort are shown. The subcohort represents the full cohort as it is a random sample of the full cohort.

BMI, body mass index; MET, metabolic equivalent; SA, South-Asian Surinamese.

**Figure 2:**
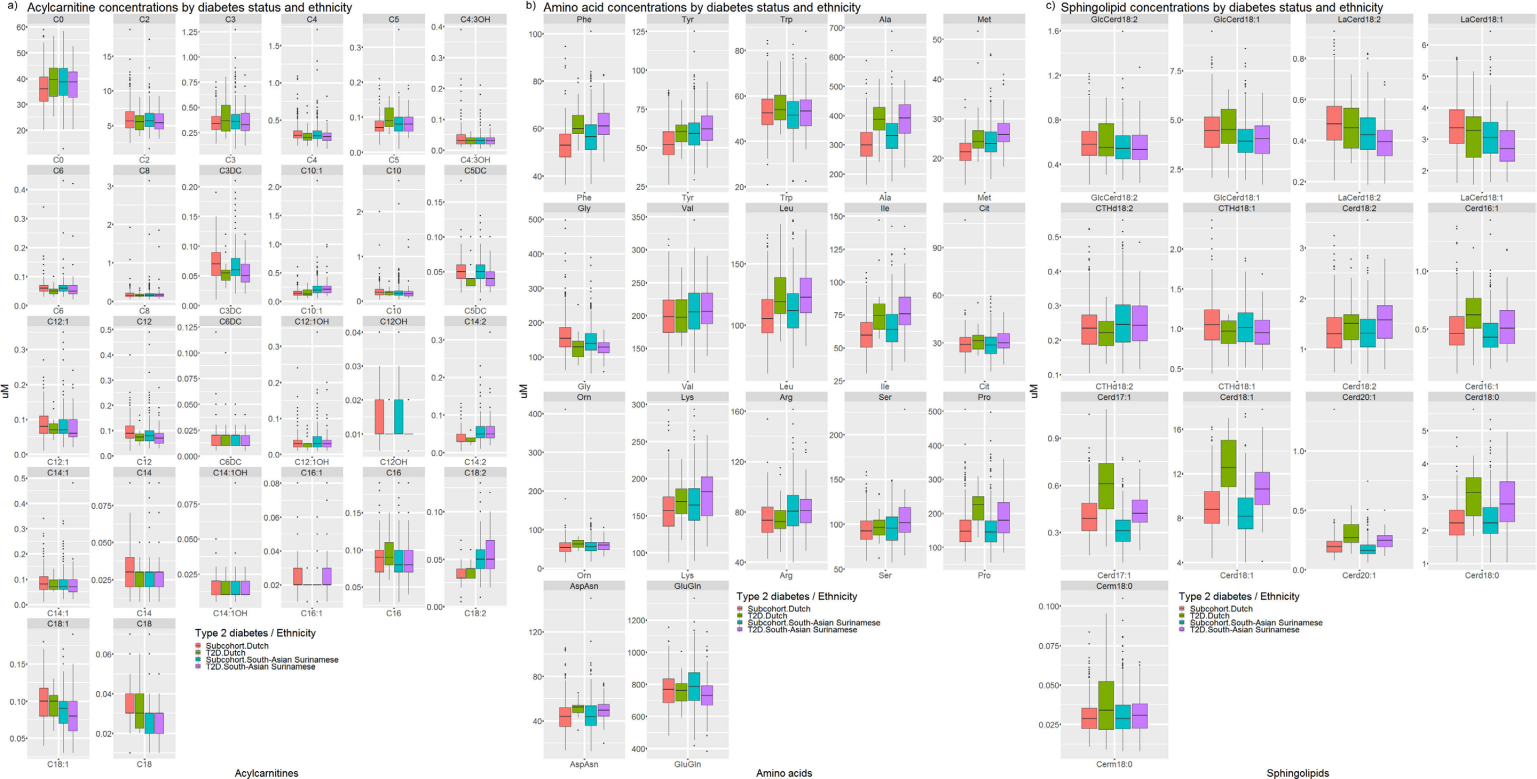
Boxplots with concentrations of (A) acylcarnitines, (B) amino acids and (C) sphingolipids in the subcohort and in diabetes cases, stratified by ethnicity. T2D, type 2 diabetes.

Baseline metabolite concentrations differed by ethnicity (figure 2; online supplementary material S3). Concentrations of most acylcarnitines were comparable between both ethnic groups. However, concentrations of carnitines C10:1, C14:2 and C18:2 were higher among South-Asian Surinamese, while concentrations of C6:DC and C18:0 were lower than in the Dutch. Larger ethnic differences were observed for amino acids. In particular, leucine, isoleucine, phenylalanine, tyrosine, alanine, methionine, glutamate and arginine were higher among South-Asian Surinamese than among Dutch participants. In addition, glycine was lower among South-Asian Surinamese in the subcohort. Most sphingolipid concentrations were either higher (the glucosylceramides, lactosylceramides and ceramides d16:1, d17:1, d18:1 and d20:1) among Dutch than South-Asian Surinamese or were comparable (globotriaosylceramides and ceramide d18:2) between both ethnic groups, except for deoxyceramide m18:0 which was lower among Dutch than South-Asian Surinamese.

### Principal component analysis

The Kaiser-Meyer-Olkin measure of sampling adequacy was 0.91, with all scores above 0.50 (range, 0.61–0.98). Moreover, the Bartlett test of sphericity showed the correlation between metabolites was sufficiently large to perform a PCA (p<0.001; online supplementary material S4). The scree plot (online supplementary material S5) showed multiple inflections, for example, at the second and fifth PCs, the first three components were retained as these reflected all included metabolites. The items that cluster on the same components suggest PC1, PC2 and PC3 represent the metabolites reflective of the acylcarnitine, amino acid and sphingolipid metabolism, respectively (online supplementary material S6). These three components could explain 43.7% of the variance in the data.

### Association between acylcarnitines, amino acids and sphingolipids with incident T2D

During a median follow-up of 3 years (IQR 2; 4), 22 participants of Dutch and 73 participants of South-Asian Surinamese ethnicity developed T2D. Characteristics and metabolite concentrations of participants who developed T2D in comparison to the randomly selected subcohort are shown in the online supplementary materials S7 and S8.

**Figure 3:**
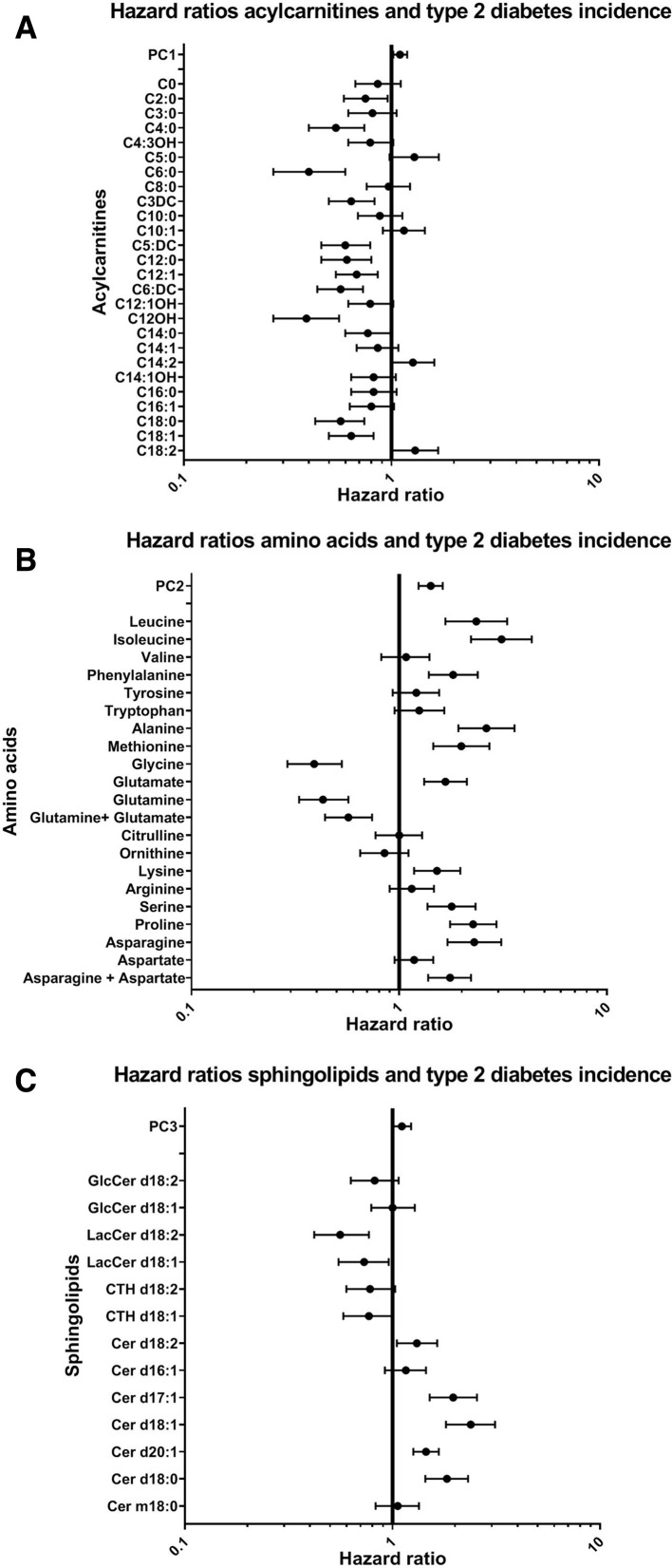
Associations between (A) acylcarnitines, (B) amino acids, and (C) sphingolipids and type 2 diabetes incidence. HRs and 95% CIs are shown. The model was adjusted for ethnicity, age, sex, smoking, physical activity, body mass index (BMI) and waist circumference.

PC1, reflective of the acylcarnitine metabolism, was positively associated with incident T2D (HR: 1.10 (95% CI 1.02 to 1.19) per 1 SD; figure 3). Because of negative factor loadings on PC1, this means that higher levels of acylcarnitines were associated with a lower risk of T2D. Factor loadings on PC2 (amino acids) and PC3 (sphingolipids) were positive, and these components were positively associated with T2D (HR: 1.42 (95% CI 1.24 to 1.62) and 1.11 (95% CI 1.01 to 1.23) per 1 SD, respectively). Thus, indicating that higher concentrations of amino acids and sphingolipids were associated with incident T2D. There was no evidence for multiplicative interactions by ethnicity (online supplementary material S9).

In line, higher concentrations of most individual acylcarnitines decreased the risk for T2D (figure 3). C5:0, C14:2 and C18:2-carnitine were, however, associated with a higher risk for T2D (eg, C18:2 HR 1.30 (95% CI 1.01 to 1.68) per 1 SD). Most individual amino acids, on the other hand, were positively associated with incident T2D (eg, isoleucine HR 3.11 (95% CI 2.22 to 4.35) per 1 SD), but in particular glycine and glutamine were negatively associated with incident T2D. Individual sphingolipids showed contrasting results. Higher levels of (dihydro)ceramides d17:1, d18:1, d20:1 and d18:0 increased the risk for T2D (eg, Cer d18:1 HR 2.38 (95% CI 1.81 to 3.12) per 1 SD), while lactosylceramides d18:2 and d18:1, on the other hand, reduced T2D risk (eg, HR 0.57 (95% CI 0.42 to 0.78) per 1 SD for lactosylceramide 18:1). CTH d18:1 and d18:2 were non-significantly associated with reduced T2D risk (HR 0.77 and 0.78 (95% CI 0.58 to 1.01 and 0.60 to 1.03)).

## Conclusions

Our study suggests that, in general, most plasma concentrations of acylcarnitines are comparable between Dutch and South-Asian Surinamese, while concentrations of amino acids were higher and concentrations of sphingolipids were lower among South-Asian Surinamese than the Dutch. Associations between metabolites and incident T2D were similar in South-Asian Surinamese and the Dutch. Higher levels of most circulating amino acids and (dihydro)ceramides were associated with higher risk for T2D, while higher levels of glycine, glutamine, lactosylceramides and most acylcarnitines were associated with lower T2D risk. Taken together, this suggests that the mechanisms underlying ethnic differences in amino acid and lactosylceramide concentrations could be investigated further to identify whether the mechanisms contribute to or predict the higher T2D risk among South-Asian Surinamese compared with Dutch.

### Acylcarnitines

Contrary to our expectations, we demonstrated a small, but negative association of acylcarnitines with incident T2D in both a set of highly correlated metabolites (PC1) and for individual acylcarnitines. Cross-sectional studies lead to the expectation that acylcarnitines inflict insulin resistance and T2D,^23–25^ though cross-sectional studies among European, Korean and Chinese populations suggested a lack in association.^26–28^ Evidence from prospective studies is limited but suggested positive associations between acylcarnitines and T2D.^7 10^ Perhaps we might have been unable to detect associations between acylcarnitines and incident T2D due to the low plasma concentrations of acylcarnitines limiting contrasts. Combining the results of these studies, it remains unclear whether elevated levels of acylcarnitines inflict insulin resistance and thereby T2D, or are a consequence of T2D.^29^

### Amino acids

The observed strengths and directions of associations are in line with previous studies on prevalent and incident T2D among various populations,^5 10 30–32^ and South Asians in particular.^5 10^ Importantly, we showed that BCAA and aromatic amino acids were associated with T2D risk, and other amino acids as well. Several mechanisms involving amino acids, and more specifically BCAAs (leucine, isoleucine and valine), have been hypothesized to cause insulin resistance and T2D. These include impaired function of branched-chain amino acid aminotransferase (BCAT) and branched-chain α-keto acid dehydrogenase (BCKDH) due to either genetic abnormalities or elevated fatty acids, proinflammatory cytokines or insulin levels leading to the accumulation of BCAAs, and also activation of the mTOR/S6K1 kinase pathway, which contributes to insulin resistance.^33^ For some amino acids, we showed an opposite association. For instance, glycine and glutamine were negatively associated with incident T2D, which is consistent with other studies.^26 32 34^ This may reflect increased gluconeogenesis,^26^ which is also observed in T2D cases.^35^ Interestingly, we did not observe the associations to differ between ethnic groups, as was suggested in previous studies. Tillin *et al* report glycine to be negatively associated with T2D in Europeans but not in South Asians (interaction p=0.05).^5^ We also did not find evidence for the reported stronger adverse association with T2D for tyrosine, alanine and phenylalanine in South Asians than in Europeans.^5^ We, therefore, propose that amino acid alterations are equally detrimental across ethnic groups.

Due to the higher plasma concentrations of most amino acids, and lower concentrations of glycine among South-Asian Surinamese, amino acids may, to some extent, contribute to the higher risk for T2D among South-Asian Surinamese. Our study adds to these previous findings that BCAAs,^5^ and a wide range of amino acids and specifically those related to reduced liver function (methionine, alanine, phenylalanine, tyrosine and lysine),^11^ may contribute to the higher risk for T2D among South-Asian Surinamese compared with Dutch. If confirmed, liver function may, therefore, be a factor to be targeted to prevent the high risk for T2D among South-Asian Surinamese.

### Sphingolipids

Our study confirmed that (dihydro)ceramides increase T2D risk.^36 37^ Several mechanisms, including β-cell apoptosis and pancreatic inflammation, were proposed to be involved in the risk posed by high levels of ceramides.^38^ However, in contrast to studies suggesting associations between deoxysphingolipids and T2D, we did not find a significant association between Cer m18:0 (also referred to as deoxydihydroceramide).^39^ This may be related to differences in analytical techniques. Importantly, we suggest that lactosylceramides are associated with a decreased T2D risk. This has not been described before, though glucosylceramides and lactosylceramides have been associated with lower fasting plasma insulin levels.^9^ Future studies are needed for confirmation and to investigate mechanisms behind this protective effect. One could speculate that bound ceramides, such as lactosylceramides, could not exert their detrimental effects. On the other hand, levels of enzymes involved in production of more complex sphingolipids could be decreased in those at risk for T2D, as was shown in genetically obese mice.^40^

Concentrations of most sphingolipids were lower among South-Asian Surinamese than Dutch, which is in line with a small-scale study by van Valkengoed *et al* with a limited set of sphingolipids.^10^ The lower plasma concentrations among South-Asian Surinamese than Dutch imply that non-oxidative pathways may not contribute to the high risk for T2D among South-Asian Surinamese compared with Dutch. Since palmitoyl-CoA is one of the rate-limiting substrates for de novo synthesis of sphingolipids,^37^ this may be related to lower intakes of saturated fatty acids among South-Asian Surinamese compared with Dutch,^41^ but may also have other causes, for example, ethnic differences in reflection of sphingolipid concentrations from individual organ compartments in plasma.

Our study is not exempt from limitations. First, although data were prospectively obtained, we cannot exclude reverse causation. Moreover, due to the short follow-up period we were unable to conduct landmark analyses to exclude the cases of diabetes diagnosed during the first years of follow-up. The observed associations between metabolites and incident T2D may either signify metabolic disturbances leading to T2D or reflect metabolic disturbances caused by an earlier stage of T2D. Second, incident T2D was determined from insurance data. Therefore, only those participants who received diabetes care were registered as T2D cases. We will have missed participants who despite having developed T2D were not diagnosed with T2D, for instance, because they did not visit their general practitioner during the period of follow-up or due to registration issues. Results should therefore be interpreted with care, especially since screening rates for T2D between ethnic groups may differ due to differences in awareness of T2D risk across ethnic groups. Awareness rates were previously shown to be higher among ethnic minority populations compared with the Dutch.^42^ Moreover, we used probabilistic data linkage to link to the Vektis database, so not all participants might be linked to the correct corresponding record. This may have led to uncertainties in T2D status. Given the more than 98% overlap of T2D statuses of participants identified in both the Achmea and the Vektis databases, however, we believe that the data linkage was of sufficient quality.

Altogether, our study suggests that dysregulation of metabolic profiles precedes incident T2D in both Dutch and South-Asian Surinamese. Increased concentrations of certain acylcarnitines, glycine, glutamine and lactosylceramides are associated with decreased T2D risk, while higher plasma concentrations of (dihydro)ceramides and most amino acids are associated with increased T2D risk. Although confirmation is needed in future studies, we suggest that disruptions of the long-chain fatty acid metabolism and increased use of non-oxidative pathways do not contribute to the higher T2D risk among South-Asian Surinamese compared with Dutch, while disturbed liver amino acid metabolism may potentially contribute. Overall, these data suggest that the disturbances underlying amino acid and sphingolipid metabolism may be predictive of T2D risk in populations of both South Asian and European background. Future studies may use our observations as starting point to unravel the underlying metabolic disturbances.
