# Supplementary material for: Contributions of amino acid, acylcarnitine and sphingolipid profiles to type 2 diabetes risk among South-Asian Surinamese and Dutch adults
Source: BMJ Open Diabetes Res Care. 2020 May 5;8(1):e001003. doi: 10.1136/bmjdrc-2019-001003 (PMC7228466; doi:10.1136/bmjdrc-2019-001003)
Supplement: Supplementary data [file bmjdrc-2019-001003supp001.pdf]

S1

We identified incident T2D cases through record linkage with two health care registrations. We first linked HELIUS data to reimbursement data from the Achmea insurance company (Achmea Health Database) with registrations from January 1, 2010 until April 30, 2016. The Achmea database contains all healthcare expenditures of every insured participant per year, including medications. From the 19,895 HELIUS participants who gave permission to link with registries and had a Citizen Service number available, we could link 15,461 participants to the Achmea data (77.7%). We used a trusted third party to link the data by Citizen Service Number, which returned the data without any identifiable variables. To define incident T2D, we considered the use of glucose lowering medication (ATC codes starting with A10) after the HELIUS inclusion date among those without T2D at inclusion.

Second, HELIUS data was linked to data registered by a business intelligence centre for healthcare (Vektis), registered from January 1, 2011 until December 31, 2017. We undertook this additional step to identify participants not insured with Achmea, increase follow-up duration, and to identify participants who received multidisciplinary care for their T2D, which is separately registered. Vektis collects insurance data of all insurance companies in the Netherlands. Linkage was done by probabilistic linkage [42] based on date of birth, sex, and postal code. We considered records to belong to the same individual if the probability of being a match was at least 95%. We chose this relatively high threshold to guarantee that the proportion of false matches in the linked data was very low. This is important because record pairs that are falsely identified as match introduce bias in the subsequent analyses of the linked data [43]. A potential downside of setting such a high threshold is that we could have missed some true matches. However, due to the large number of observations in both datasets, we considered this as less important.

In some cases (n=14) a HELIUS participant was assigned multiple matches, and then T2D status was set to missing. We censored participants after loss to follow-up. Of the 20,681 HELIUS participants who gave permission to link with registries, we linked 18,425 participants with Vektis data (89%). To define incident T2D, we considered either receiving multidisciplinary care based on care performance codes or use of pharmacological medication for diabetes.

In total, we were able to link 96.4% of all the HELIUS participants who had provided permission for data linkage (n=19,932 of 20,681) with at least one of two data sources. Finally, we defined incident T2D as a registration of one of the considered codes in either the Achmea database or the Vektis database.

Follow-up duration was determined from inclusion date within HELIUS until the latest moment of data linkage, or the moment that a participant developed T2D. For participants who developed T2D, the first date of registration with T2D in either the Achmea or the Vektis database was used to determine follow-up duration.

S2. All sphingolipid species were measured as their corresponding ''lyso'' derivates yielded upon microwave-assisted deacylation.

Compound	MRM (m/z)	Cone voltage (V)	Collision voltage (V)	Retention time (min)	Calibration curve	Internal standard
**1-Deoxysphinganine(m18:0)**	286.5 > 268.5	25	15	3.26	1-Deoxysphinganine(m18:0)	d5-Glucosylsphingosine(d18:1)
**Sphinganine(d18:0)**	302.3 > 284.3	25	15	3.22	Sphinganine(d18:0)	d5-Glucosylsphingosine(d18:1)
**Sphingosine(d18:1)**	300.3 > 282.3	20	10	3.17	Sphingosine(d18:1)	d5-Glucosylsphingosine(d18:1)
**Sphingosine(d18:2)**	298.3 > 280.3	20	10	3.06	Sphingosine(d18:1)	d5-Glucosylsphingosine(d18:1)
**Sphingosine(d16:1)**	272.3 > 254.3	15	10	3.02	Sphingosine(d18:1)	d5-Glucosylsphingosine(d18:1)
**Sphingosine(d17:1)**	286.3 > 268.3	15	10	3.10	Sphingosine(d18:1)	d5-Glucosylsphingosine(d18:1)
**Sphingosine(d20:1)**	328.3 > 310.3	20	10	3.31	Sphingosine(d18:1)	d5-Glucosylsphingosine(d18:1)
**Glucosylsphingosine(d18:1)**	462.4 > 282.3	25	20	3.12	Glucosylsphingosine(d18:1)	d5-Glucosylsphingosine(d18:1)
**Glucosylsphingosine(d18:2)**	460.4 > 280.3	25	20	3.01	Glucosylsphingosine(d18:1)	d5-Glucosylsphingosine(d18:1)
**Lactosylsphingosine(d18:1)**	624.4 > 282.3	35	30	3.10	Lactosylsphingosine(d18:1)	d5-Glucosylsphingosine(d18:1)
**Lactosylsphingosine(d18:2)**	622.4 > 280.3	35	30	2.98	Lactosylsphingosine(d18:1)	d5-Glucosylsphingosine(d18:1)
**Globotriaosylsphingosine(d18:1)**	786.5 > 282.3	45	35	3.08	Globotriaosylsphingosine(d18:1)	d5-Glucosylsphingosine(d18:1)
**Globotriaosylsphingosine(d18:2)**	784.5 > 287.4	45	35	2.96	Globotriaosylsphingosine(d18:1)	d5-Glucosylsphingosine(d18:1)
**d5-Glucosylsphingosine(d18:1)**	467.4 > 291.5	25	20	3.12	Not applicable	Not applicable

S3: Metabolite concentrations (μmol/l) in the subcohort representative of the entire study population.

	Total subcohort (n=677)	Dutch subcohort (n=334)	SA subcohort (n=343)
**Acylcarnitines**			
**Mean PC 1**	-0.01 (3.61)	0.04 (3.70)	-0.06 (3.51)
**Mean C0**	37.3 (7.4)	36.0 (7.1)	38.6 (7.4)
**Median C2:0**	5.7 (4.7-6.9)	5.7 (4.7-7.0)	5.7 (4.8-6.8)
**Median C3:0**	0.34 (0.28-0.43)	0.34 (0.28-0.41)	0.36 (0.29-0.43)
**Median C4:0**	0.30 (0.25-0.36)	0.30 (0.26-0.36)	0.29 (0.25-0.36)
**Median C4:3OH**	0.03 (0.02-0.04)	0.03 (0.02-0.05)	0.03 (0.02-0.04)
**Median C5:0**	0.08 (0.06-0.10)	0.07 (0.06-0.09)	0.08 (0.06-0.10)
**Median C6:0**	0.06 (0.05-0.07)	0.06 (0.05-0.07)	0.06 (0.05-0.07)
**Median C8:0**	0.16 (0.12-0.20)	0.15 (0.12-0.21)	0.16 (0.13-0.20)
**Median C3DC**	0.07 (0.05-0.08)	0.07 (0.05-0.09)	0.06 (0.05-0.08)
**Median C10:0**	0.18 (0.14-0.25)	0.19 (0.14-0.26)	0.17 (0.13-0.24)
**Median C10:1**	0.17 (0.12-0.23)	0.14 (0.11-0.18)	0.20 (0.15-0.27)
**Median C5:DC**	0.05 (0.04-0.06)	0.05 (0.04-0.06)	0.05 (0.04-0.06)
**Median C12:0**	0.08 (0.07-0.11)	0.09 (0.07-0.12)	0.08 (0.06-0.10)
**Median C12:1**	0.08 (0.06-0.11)	0.08 (0.06-0.11)	0.07 (0.06-0.10)
**Median C6:DC**	0.02 (0.01-0.02)	0.02 (0.01-0.02)	0.02 (0.01-0.02)
**Median C12:1OH**	0.03 (0.02-0.05)	0.03 (0.02-0.04)	0.03 (0.02-0.05)
**Median C12OH**	0.01 (0.01-0.02)	0.01 (0.01-0.02)	0.01 (0.01-0.02)
**Median C14:0**	0.03 (0.02-0.03)	0.03 (0.02-0.04)	0.03 (0.02-0.03)
**Median C14:1**	0.07 (0.06-0.10)	0.08 (0.06-0.11)	0.07 (0.06-0.10)
**Median C14:2**	0.04 (0.03-0.06)	0.03 (0.03-0.05)	0.05 (0.04-0.07)

**Median C14:1OH**	0.01 (0.01-0.02)	0.02 (0.01-0.02)	0.01 (0.01-0.02)
**Mean C16:0**	0.09 (0.02)	0.09 (0.03)	0.09 (0.02)
**Median C16:1**	0.02 (0.01)	0.02 (0.02-0.03)	0.02 (0.02-0.02)
**Mean C18:0**	0.03 (0.01)	0.03 (0.01)	0.03 (0.01)
**Mean C18:1**	0.09 (0.03)	0.10 (0.03)	0.09 (0.02)
**Mean C18:2**	0.04 (0.02)	0.04 (0.01)	0.05 (0.02)
**Amino acids**			
**Mean PC 2**	0.01 (2.58)	-0.63 (2.33)	0.63 (2.66)
**Mean Leucine**	110.6 (21.3)	107.6 (20.0)	113.6 (22.1)
**Mean Isoleucine**	63.2 (15.6)	60.7 (14.5)	65.7 (16.3)
**Mean Valine**	203.7 (37.4)	199.8 (36.4)	207.4 (38.0)
**Median Phenylalanine**	54.7 (8.4)	52.9 (48.0-57.8)	56.6 (51.2-61.7)
**Mean Tyrosine**	56.8 (12.4)	53.7 (11.1)	59.9 (12.9)
**Mean Tryptophan**	52.3 (8.9)	52.9 (8.7)	51.8 (9.0)
**Mean Alanine**	320.3 (70.9)	306.0 (65.8)	334.1 (73.0)
**Median Methionine**	22.7 (4.0)	21.7 (19.4-23.9)	24.3 (21.6-26.7)
**Median Glycine**	147.0 (50.9)	153.7 (128.7-185.9)	139.4 (120.4-168.5)
**Mean Glutamine**	569.0 (97.3)	572.3 (96.7)	568.5 (98.0)
**Median Glutamate**	194.6 (72.7)	194.6 (152.2-224.2)	224.3 (169.7-255.8)
**Mean Glutamine + Glutamate**	778.7 (127.2)	765.8 (118.1)	789.8 (134.0)
**Median Citrulline**	28.7 (23.7-33.6)	28.9 (24.1-33.5)	28.5 (23.2-33.6)
**Median Ornithine**	54.5 (44.7-67.1)	53.9 (43.5-66.7)	55.5 (45.8-67.4)
**Mean Lysine**	162.4 (33.2)	158.1 (31.6)	166.6 (34.2)
**Mean Arginine**	78.1 (17.1)	74.3 (14.5)	81.8 (18.5)

**Mean Serine**	95.3 (17.7)	94.6 (17.0)	95.9 (18.4)
**Median Proline**	146.0 (116.2-178.9)	146.7 (116.8-181.0)	145.3 (116.0-178.1)
**Median Asparagine**	23.4 (18.9-28.7)	23.1 (18.5-29.2)	23.7 (19.1-28.5)
**Median Aspartate**	19.3 (13.7-25.3)	18.9 (14.2-24.6)	19.7 (13.4-26.6)
**Median Asparagine + Aspartate**	43.7 (34.9-52.4)	43.8 (34.8-51.9)	43.7 (35.9-53.5)
**Sphingolipids**			
**PC3**	0.02 (2.43)	0.55 (2.44)	-0.50 (2.31)
**Mean GlcCer d18:2**	0.58 (0.17)	0.60 (0.17)	0.56 (0.16)
**Mean GlcCer d18:1**	4.23 (1.11)	4.47 (1.16)	4.01 (1.01)
**Mean LacCer d18:2**	0.47 (0.12)	0.49 (0.13)	0.44 (0.11)
**Mean LacCer d18:1**	3.27 (0.78)	3.45 (0.79)	3.10 (0.75)
**Mean CTH d18:2**	0.25 (0.08)	0.24 (0.07)	0.25 (0.08)
**Mean CTH d18:1**	1.05 (0.28)	1.08 (0.29)	1.03 (0.26)
**Mean Cer d18:2**	1.37 (0.43)	1.36 (0.44)	1.35 (0.42)
**Mean Cer d16:1**	0.48 (0.19)	0.50 (0.20)	0.46 (0.18)
**Mean Cer d17:1**	0.36 (0.13)	0.41 (0.13)	0.32 (0.11)
**Mean Cer d18:1**	8.78 (2.19)	9.08 (2.29)	8.48 (2.04)
**Mean Cer d20:1**	0.19 (0.08)	0.20 (0.09)	0.18 (0.07)
**Mean Cer d18:0**	2.31 (0.62)	2.27 (0.58)	2.35 (0.66)
**Mean Cer m18:0**	0.03 (0.01)	0.03 (0.01)	0.03 (0.01)

Data are mean (SD) or median (IQR). SA = South-Asian Surinamese

S4: Correlation matrix metabolites

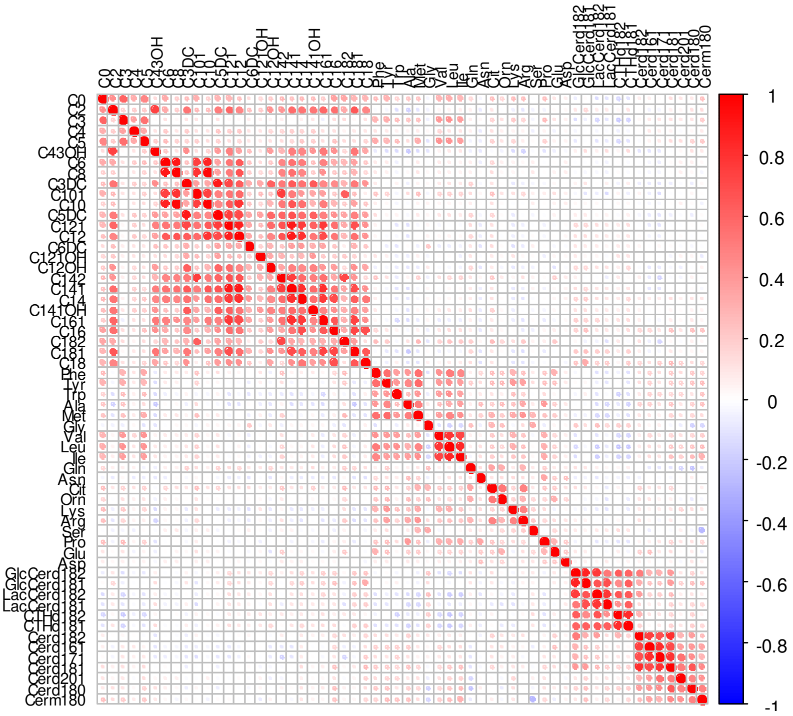

S5: Scree plot

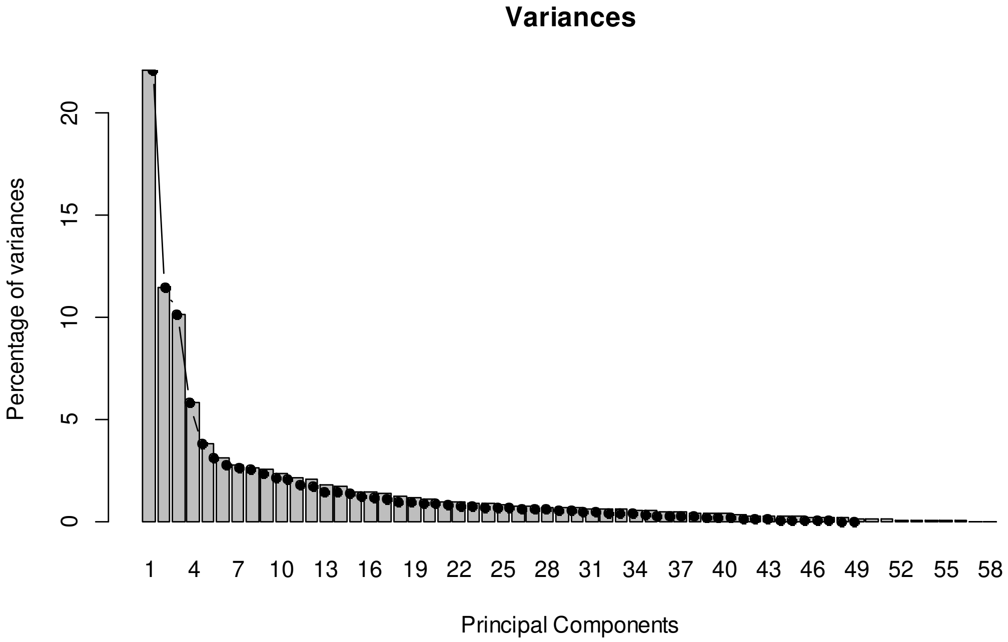

S6: Factor loadings of PCA in the subcohort representative of the entire study population.

Acylcarnitine	PC1	PC2	PC3	Amino acid	PC1	PC2	PC3	Sphingolipid	PC1	PC2	PC3
C0	-0.39	**0.54**		Leucine		**0.75**		GlcCer d18:2	-0.13		**0.75**
C2	**-0.75**			Isoleucine		**0.69**		GlcCer d18:1	-0.15		**0.74**
C3	-0.14	**0.68**		Valine		**0.69**		LacCer d18:2			**0.70**
C4	-0.22	**0.40**		Phenylalanine		**0.57**		LacCer d18:1			**0.60**
C4:3OH	**-0.64**			Tyrosine		**0.53**		CTH d18:2			**0.54**
C5	-0.26	**0.60**		Tryptophan		**0.50**		CTH d18:1	-0.12		**0.60**
C6	**-0.71**	0.22		Alanine	0.15	**0.44**		Cer d18:2			**0.74**
C8	**-0.80**			Methionine		**0.51**		Cer d16:1	0.11		**0.67**
C3DC	**-0.80**	0.11		Glycine		-0.21	0.14	Cer d17:1			**0.72**
C10:1	**-0.69**	0.19	-0.19	Glutamine	-0.13			Cer d18:1			**0.82**
C10	**-0.82**			Asparagine			-0.11	Cer d20:1			**0.46**
C5:DC	**-0.81**	0.13		Citrulline	-0.17	0.23	0.11	Cer d18:0			**0.56**
C12:1	**-0.93**			Ornithine		0.24		Cer m18:0			**0.45**
C6:DC	**-0.44**			Lysine		0.34					
C12:1OH	**-0.51**			Arginine		0.24					
C12OH	**-0.60**			Serine		-0.20					
C14:2	**-0.77**		-0.13	Proline		**0.41**					
C14:1	**-0.93**			Glutamate		0.13					
C14	**-0.81**		0.19	Aspartate							
C14:1OH	**-0.72**										
C16:1	**-0.76**	-0.11	0.15								
C16	**-0.67**	0.21	0.28								

C18:2	**-0.49**	0.18	-0.10
C18:1	**-0.76**		0.17
C18	**-0.55**	0.10	0.36

Factor loadings >0.40 are marked in bold. Factor loadings <0.10 are not shown.

S7: Characteristics in subcohort and cases, stratified by ethnicity

	Dutch subcohort (n=334)	SA subcohort (n=343)	Dutch T2D (n=22)	SA T2D (n=73)
**Mean age (years)**	46.0 (13.8)	44.0 (12.9)	59.2 (9.0)	53.0 (9.4)
**% Male (*n*)**	49.4 (165)	44.0 (151)	68.2 (15)	37.0 (27)
**Median packyears of smoking**	1.2 (0.0; 10.0)	0 (0; 2.8)	15.6 (4.9; 29.4)	0 (0; 1.5)
**Mean physical activity (met/week)**	2812 (1312)	2751 (1802)	2357 (1176)	2713 (1690)
**Mean BMI (kg/m^2)**	24.4 (3.8)	25.7 (4.4)	27.9 (2.9)	28.3 (4.2)
**Mean waist circumference (cm)**	88.9 (12.2)	90.0 (12.3)	101.4 (7.6)	97.1 (9.2)
**Socio-economic status (%)**				
Never been to school / Elementary schooling only	4.2 (14)	10.5 (36)	9.1 (2)	21.9 (16)
Lower vocational schooling or lower secondary schooling	10.8 (36)	32.7 (112)	31.8 (7)	39.7 (29)
Intermediate vocational schooling or intermediate/higher secondary schooling	21.9 (73)	30.3 (104)	22.7 (5)	26.0 (19)
Higher vocational schooling or university	63.2 (211)	26.5 (91)	36.4 (8)	12.3 (9)

Data are mean (SD) or median (IQR). SA = South-Asian Surinamese.

S8: Metabolite concentrations (µmol/l) in subcohort and cases, stratified by ethnicity

	Dutch subcohort (n=334)	SA subcohort (n=343)	Dutch T2D (n=22)	SA T2D (n=73)
**Acylcarnitines**				
**Mean PC 1**	0.10 (3.63)	-0.11 (3.54)	0.58 (2.80)	0.64 (3.79)
**Mean C0**	36.0 (7.1)	38.6 (7.4)	38.7 (7.7)	37.3 (7.6)
**Median C2:0**	5.7 (4.7-7.0)	5.7 (4.8-6.8)	5.5 (4.4-6.5)	5.4 (4.5-6.7)
**Median C3:0**	0.35 (0.28-0.41)	0.36 (0.29-0.43)	0.37 (0.26-0.52)	0.33 (0.27-0.44)
**Median C4:0**	0.30 (0.26-0.36)	0.29 (0.25-0.36)	0.27 (0.24-0.33)	0.28 (0.23-0.33)
**Median C4:3OH**	0.03 (0.02-0.05)	0.03 (0.02-0.04)	0.03 (0.02-0.04)	0.03 (0.02-0.04)
**Median C5:0**	0.07 (0.06-0.09)	0.08 (0.06-0.10)	0.09 (0.07-0.13)	0.08 (0.06-0.10)
**Median C6:0**	0.06 (0.05-0.07)	0.06 (0.05-0.07)	0.05 (0.04-0.06)	0.05 (0.04-0.07)
**Median C8:0**	0.15 (0.12-0.21)	0.16 (0.13-0.20)	0.15 (0.13-0.19)	0.16 (0.12-0.19)
**Median C3DC**	0.07 (0.05-0.09)	0.06 (0.05-0.08)	0.06 (0.04-0.06)	0.05 (0.04-0.07)
**Median C10:0**	0.19 (0.14-0.26)	0.17 (0.13-0.24)	0.19 (0.14-0.22)	0.16 (0.12-0.22)
**Median C10:1**	0.14 (0.11-0.18)	0.20 (0.15-0.27)	0.13 (0.10-0.20)	0.21 (0.15-0.27)
**Median C5:DC**	0.05 (0.04-0.06)	0.05 (0.04-0.06)	0.04 (0.03-0.04)	0.04 (0.03-0.05)
**Median C12:0**	0.09 (0.07-0.12)	0.08 (0.06-0.10)	0.08 (0.06-0.09)	0.07 (0.05-0.09)
**Median C12:1**	0.08 (0.06-0.11)	0.07 (0.06-0.10)	0.07 (0.06-0.09)	0.06 (0.05-0.10)
**Median C6:DC**	0.02 (0.01-0.02)	0.02 (0.01-0.02)	0.02 (0.01-0.02)	0.01 (0.01-0.02)
**Median C12:1OH**	0.03 (0.02-0.04)	0.03 (0.02-0.05)	0.03 (0.02-0.03)	0.03 (0.02-0.04)
**Median C12OH**	0.01 (0.01-0.02)	0.01 (0.01-0.02)	0.01 (0.01-0.01)	0.01 (0.01-0.01)
**Median C14:0**	0.03 (0.02-0.04)	0.03 (0.02-0.03)	0.03 (0.02-0.03)	0.03 (0.02-0.03)
**Median C14:1**	0.08 (0.06-0.11)	0.07 (0.06-0.10)	0.07 (0.06-0.10)	0.07 (0.05-0.10)

**Median C14:2**	0.03 (0.03-0.05)	0.05 (0.04-0.07)	0.03 (0.03-0.04)	0.05 (0.04-0.07)
**Median C14:1OH**	0.02 (0.01-0.02)	0.01 (0.01-0.02)	0.01 (0.01-0.02)	0.01 (0.01-0.02)
**Mean C16:0**	0.09 (0.03)	0.09 (0.02)	0.09 (0.02)	0.08 (0.03)
**Median C16:1**	0.02 (0.02-0.03)	0.02 (0.02-0.02)	0.02 (0.02-0.02)	0.02 (0.02-0.03)
**Mean C18:0**	0.03 (0.01)	0.03 (0.01)	0.03 (0.01)	0.03 (0.01)
**Mean C18:1**	0.10 (0.03)	0.09 (0.02)	0.10 (0.02)	0.08 (0.03)
**Mean C18:2**	0.04 (0.01)	0.05 (0.02)	0.04 (0.01)	0.06 (0.02)
**Amino acids**				
**Mean PC 2**	-0.67 (2.25)	0.67 (2.55)	1.87 (1.79)	2.10 (2.41)
**Mean Leucine**	107.6 (20.0)	113.6 (22.1)	123.0 (22.2)	125.6 (22.4)
**Mean Isoleucine**	60.7 (14.5)	65.7 (16.3)	78.0 (20.7)	79.1 (19.7)
**Mean Valine**	199.8 (36.4)	207.4 (38.0)	202.5 (31.9)	211.2 (32.8)
**Median Phenylalanine**	52.9 (48.0-57.8)	56.6 (51.2-61.7)	59.9 (57.8-65.6)	61.1 (57.4-66.4)
**Mean Tyrosine**	53.7 (11.1)	59.9 (12.9)	60.7 (11.0)	62.4 (12.3)
**Mean Tryptophan**	52.9 (8.7)	51.8 (9.0)	55.5 (8.2)	53.3 (10.3)
**Mean Alanine**	306.0 (65.8)	334.1 (73.0)	392.6 (67.8)	387.1 (69.8)
**Median Methionine**	21.7 (19.4-23.9)	24.3 (21.6-26.7)	24.1 (22.6-27.1)	26.8 (24.2-28.8)
**Median Glycine**	153.7 (128.7-185.9)	139.4 (120.4-168.5)	128.9 (100.2-145.7)	127.7 (111.8-142.5)
**Mean Glutamine**	572.3 (96.7)	568.5 (98.0)	513.7 (80.9)	496.3 (104.2)
**Median Glutamate**	194.6 (152.2-224.2)	224.3 (169.7-255.8)	243.7 (231.4-252.9)	237.5 (214.5-260.3)
**Mean Glutamine + Glutamate**	765.8 (118.1)	789.8 (134.0)	756.9 (92.8)	733.9 (130.9)
**Median Citrulline**	28.9 (24.1-33.5)	28.5 (23.2-33.6)	31.3 (26.0-34.8)	30.0 (26.6-35.9)
**Median Ornithine**	53.9 (43.5-66.7)	55.5 (45.8-67.4)	63.0 (54.5-72.8)	59.8 (48.2-67.5)

**Mean Lysine**	158.1 (31.6)	166.6 (34.2)	170.8 (27.5)	178.9 (34.8)
**Mean Arginine**	74.3 (14.5)	81.8 (18.5)	75.0 (17.1)	82.5 (17.3)
**Mean Serine**	94.6 (17.0)	95.9 (18.4)	97.8 (17.3)	105.8 (22.7)
**Median Proline**	146.7 (116.8-181.0)	145.3 (116.0-178.1)	225.3 (180.9-249.8)	179.71(142.6-232.8)
**Median Asparagine**	23.1 (18.5-29.2)	23.7 (19.1-28.5)	29.4 (26.9-35.1)	29.8 (25.5-34.0)
**Median Aspartate**	18.9 (14.2-24.6)	19.7 (13.4-26.6)	19.4 (17.2-23.9)	18.8 (16.4-22.4)
**Median Asparagine + Aspartate**	43.8 (34.8-51.9)	43.7 (35.9-53.5)	52.2 (47.4-54.3)	49.6 (44.4-54.8)
**Sphingolipids**				
**PC3**	0.55 (2.44)	-0.50 (2.31)		
**Mean GlcCerd182 (nM)**	599.6 (174.6)	558.7 (161.5)	601.1 (191.9)	553.8 (173.6)
**Mean GlcCerd181 (nM)**	4466 (1162)	4005 (1009)	4587 (1139)	3990 (1031)
**Mean LacCerd182 (nM)**	490.8 (129.1)	442.0 (114.1)	461.3 (112.1)	393.5 (100.2)
**Mean LacCerd181 (nM)**	3452 (788.9)	3096 (745.8)	3205 (991.5)	2740 (646.0)
**Mean CTHd182 (nM)**	239.7 (72.2)	254.9 (79.4)	223.4 (47.8)	254.0 (75.7)
**Mean CTHd181 (nM)**	1075.7 (293.3)	1033.3 (260.7)	953.9 (177.0)	965.8 (243.3)
**Mean Cerd182 (nM)**	1364.2 (441.7)	1349.4 (420.8)	1552.2 (544.6)	1582.7 (490.3)
**Mean Cerd161 (nM)**	501.0 (200.4)	462.0 (182.4)	615.2 (251.0)	542.4 (206.4)
**Mean Cerd171 (nM)**	406.1 (133.9)	324.6 (111.0)	609.6 (233.1)	438.5 (127.7)
**Mean Cerd181 (nM)**	9080 (2294)	8480 (2040)	12672 (2861)	10743 (2469)
**Mean Cerd201 (nM)**	200.5 (90.4)	180.2 (73.0)	291.6 (101.9)	243.5 (68.7)
**Mean Cerd180 (nM)**	2272 (580.2)	2350 (659.5)	3119 (918.2)	2907 (816.5)
**Mean Cerm180 (nM)**	30.5 (11.1)	37.2 (21.9)	31.4 (13.6)	32.5 (13.4)

Data are mean (SD) or median (IQR). SA = South-Asian Surinamese.

S9: Association of metabolites with incident type 2 diabetes, stratified by ethnicity

	HR (95%-CI)	p-value	HR (95%-CI)	p-value	p-value interaction
**Acylcarnitines**					
**PC1**	1.11 (0.97; 1.28)	0.14	1.10 (1.00; 1.21)	***0.04***	0.86
**C0**	1.06 (0.60; 1.88)	0.84	0.76 (0.56; 1.04)	0.08	0.21
**C2:0**	0.70 (0.44; 1.13)	0.14	0.75 (0.54; 1.03)	0.08	0.97
**C3:0**	0.86 (0.47; 1.58)	0.63	0.78 (0.56; 1.07)	0.12	0.62
**C4:0**	0.44 (0.23; 0.86)	***0.02***	0.59 (0.41; 0.85)	***0.005***	0.60
**C4:3OH**	0.73 (0.44; 1.22)	0.23	0.84 (0.61; 1.14)	0.26	0.81
**C5:0**	1.77 (0.98; 3.20)	0.06	1.21 (0.88; 1.65)	0.24	0.19
**C6:0**	0.14 (0.05; 0.43)	***0.001****	0.43 (0.27; 0.69)	***0.001****	0.15
**C8:0**	0.90 (0.51; 1.60)	0.72	0.95 (0.71; 1.27)	0.70	0.86
**C3DC**	0.67 (0.40; 1.10)	0.11	0.61 (0.45; 0.85)	***0.003***	0.64
**C10:0**	0.89 (0.52; 1.53)	0.68	0.89 (0.66; 1.18)	0.41	0.97
**C10:1**	1.01 (0.56; 1.79)	0.99	0.84 (0.61; 1.15)	0.28	0.54
**C5:DC**	0.43 (0.24; 0.76)	***0.004***	0.63 (0.46; 0.87)	***0.004***	0.36
**C12:0**	0.52 (0.30; 0.89)	***0.02***	0.61 (0.43; 0.88)	***0.007***	0.87
**C12:1**	0.65 (0.41; 1.02)	0.06	0.69 (0.51; 0.93)	***0.02***	0.95
**C6:DC**	0.67 (0.41; 1.09)	0.11	0.57 (0.41; 0.78)	***0.001****	0.50
**C12:1OH**	0.66 (0.39; 1.12)	0.12	0.75 (0.55; 1.01)	0.06	0.81
**C12OH**	0.35 (0.19; 0.67)	***0.001***	0.40 (0.25; 0.62)	***<0.001****	0.79
**C14:0**	0.61 (0.37; 1.00)	0.05	0.87 (0.64; 1.18)	0.36	0.39
**C14:1**	0.86 (0.53; 1.39)	0.54	0.86 (0.64; 1.16)	0.32	0.94
**C14:2**	1.02 (0.62; 1.68)	0.93	1.00 (0.72; 1.37)	1.00	0.91

**C14:1OH**	0.64 (0.37; 1.09)	0.10	0.85 (0.64; 1.13)	0.26	0.47
**C16:0**	0.86 (0.53; 1.41)	0.55	0.80 (0.59; 1.09)	0.16	0.49
**C16:1**	0.65 (0.40; 1.04)	0.07	0.90 (0.66; 1.23)	0.52	0.34
**C18:0**	0.66 (0.37; 1.15)	0.14	0.58 (0.41; 0.82)	***0.002***	0.35
**C18:1**	0.77 (0.48; 1.23)	0.28	0.63 (0.46; 0.87)	***0.005***	0.42
**C18:2**	1.24 (0.63; 2.45)	0.53	0.87 (0.62; 1.24)	0.44	0.31
**Amino acids**					
**PC2**	1.97 (1.42; 2.73)	***<0.001***	1.36 (1.16; 1.58)	***<0.001***	0.05
**Leucine**	2.32 (1.16; 4.65)	***0.02***	2.26 (1.49; 3.42)	***<0.001****	0.91
**Isoleucine**	3.73 (1.78; 7.79)	***0.001****	2.84 (1.92; 4.21)	***<0.001****	0.68
**Valine**	0.85 (0.51; 1.41)	0.53	1.06 (0.77; 1.46)	0.75	0.66
**Phenylalanine**	2.62 (1.55; 4.40)	***<0.001****	1.58 (1.15; 2.17)	***0.005***	0.05
**Tyrosine**	1.34 (0.75; 2.40)	0.33	1.02 (0.74; 1.40)	0.92	0.32
**Tryptophan**	1.77 (0.95; 3.30)	0.07	1.26 (0.90; 1.76)	0.19	0.48
**Alanine**	4.70 (2.05; 10.79)	***0.001****	2.19 (1.53; 3.15)	***<0.001****	0.13
**Methionine**	2.66 (1.32; 5.35)	***0.01***	1.73 (1.20; 2.48)	***0.003****	0.17
**Glycine**	0.25 (0.11; 0.55)	***0.001****	0.47 (0.33; 0.67)	***<0.001****	0.13
**Glutamate**	2.66 (1.49; 4.73)	***0.001****	1.32 (1.01; 1.73)	***0.05***	***0.04***
**Glutamine**	0.33 (0.17; 0.62)	***0.001****	0.40 (0.29; 0.55)	***<0.001****	0.92
**Glutamine+ Glutamate**	0.71 (0.39; 1.27)	0.25	0.51 (0.38; 0.69)	***<0.001****	0.25
**Citrulline**	1.02 (0.56; 1.84)	0.96	1.00 (0.74; 1.34)	0.98	0.93
**Ornithine**	1.06 (0.62; 1.81)	0.84	0.79 (0.58; 1.08)	0.14	0.18
**Lysine**	1.68 (0.97; 2.89)	0.06	1.34 (0.98; 1.82)	0.07	0.62

**Arginine**	1.17 (0.66; 2.09)	0.59	0.98 (0.73; 1.31)	0.89	0.87
**Serine**	1.49 (0.84; 2.63)	0.17	1.85 (1.35; 2.55)	***<0.001****	0.30
**Proline**	3.37 (1.81; 6.26)	***<0.001****	2.15 (1.58; 2.94)	***<0.001****	0.35
**Asparagine**	4.83 (2.34; 9.94)	***<0.001****	2.13 (1.53; 2.96)	***<0.001****	0.08
**Aspartate**	1.10 (0.69; 1.77)	0.69	1.24 (0.97; 1.60)	0.09	0.60
**Asparagine + Aspartate**	2.02 (1.20; 3.42)	***0.008***	1.76 (1.33; 2.33)	***<0.001****	0.74
**PC3**	1.14 (0.93; 1.41)	0.22	1.11 (0.99; 1.24)	0.08	0.71
**GlcCer d18:2**	0.74 (0.44; 1.25)	0.26	0.83 (0.60; 1.14)	0.25	0.81
**GlcCer d18:1**	0.91 (0.58; 1.43)	0.68	1.03 (0.77; 1.37)	0.87	0.76
**LacCer d18:2**	0.67 (0.39; 1.15)	0.15	0.50 (0.34; 0.73)	***0.001****	0.40
**LacCer d18:1**	0.77 (0.46; 1.28)	0.31	0.68 (0.48; 0.96)	***0.03***	0.71
**CTH d18:2**	0.69 (0.38; 1.24)	0.22	0.80 (0.59; 1.10)	0.17	0.51
**CTH d18:1**	0.70 (0.43; 1.14)	0.15	0.78 (0.56; 1.10)	0.17	0.60
**Cer d18:2**	1.03 (0.66; 1.60)	0.90	1.41 (1.08; 1.84)	***0.01***	0.28
**Cer d16:1**	1.02 (0.68; 1.54)	0.92	1.22 (0.92; 1.63)	0.16	0.75
**Cer d17:1**	1.83 (1.18; 2.83)	***0.007***	2.28 (1.64; 3.18)	***<0.001****	0.32
**Cer d18:1**	2.71 (1.57; 4.68)	***0.001****	2.36 (1.72; 3.25)	***<0.001****	0.61
**Cer d20:1**	1.39 (1.13; 1.72)	***0.002****	1.55 (1.20; 2.02)	***0.001****	0.41
**Cer d18:0**	2.96 (1.62; 5.41)	***<0.001****	1.70 (1.31; 2.20)	***<0.001****	0.12
**Cer m18:0**	1.28 (0.73; 2.25)	0.39	1.01 (0.77; 1.32)	0.94	0.31

P-values <0.05 were considered statistically significant and are marked in bold italic. P-value interaction ethnicity shows the p-values for the multiplicative interaction between metabolites and ethnicity with type 2 diabetes incidence as the outcome. HRs were Prentice weighted and shown for ethnic groups combined, as no evidence for interaction by ethnicity was observed, and stratified by ethnicity. The fully adjusted model was adjusted for age, sex, smoking, physical activity, BMI and waist circumference. *HRs were still statistically significant (p<0.05) after holm-adjustment for multiple testing of individual metabolites.
